# MiR-378a-3p Is Critical for Burkitt Lymphoma Cell Growth

**DOI:** 10.3390/cancers12123546

**Published:** 2020-11-27

**Authors:** Fubiao Niu, Agnieszka Dzikiewicz-Krawczyk, Jasper Koerts, Debora de Jong, Laura Wijenberg, Margot Fernandez Hernandez, Izabella Slezak-Prochazka, Melanie Winkle, Wierd Kooistra, Tineke van der Sluis, Bea Rutgers, Miente Martijn Terpstra, Klaas Kok, Joost Kluiver, Anke van den Berg

**Affiliations:** 1 Departments of Pathology and Medical Biology, University Medical Center Groningen, University of Groningen, 9700 RB Groningen, The Netherlands; f.niu@umcg.nl (F.N.); j.a.koerts@umcg.nl (J.K.); d.de.jong03@umcg.nl (D.d.J.); l.wijenberg@student.rug.nl (L.W.); margot.fernandezhz@udlap.mx (M.F.H.); m.winkle@protonmail.com (M.W.); w.kooistra@umcg.nl (W.K.); t.van.der.sluis@umcg.nl (T.v.d.S.); b.rutgers@umcg.nl (B.R.); j.l.kluiver@umcg.nl (J.K.); 2 Institute of Human Genetics, Polish Academy of Sciences, 60-479 Poznan, Poland; krawczyk@man.poznan.pl; 3 Biotechnology Centre, Silesian University of Technology, 44-100 Gliwice, Poland; izabella.slezak-prochazka@polsl.pl; 4 Department of Genetics, University Medical Center Groningen, University of Groningen, 9700 RB Groningen, The Netherlands; m.m.terpstra@umcg.nl (M.M.T.); k.kok@umcg.nl (K.K.)

**Keywords:** Burkitt lymphoma, miR-378a-3p, cell growth, microRNA

## Abstract

**Simple Summary:**

MicroRNAs (miRNAs) are small RNAs that regulate expression of specific target genes. We observed elevated levels of miR-378a-3p in Burkitt lymphoma (BL) and studied its role in the pathogenesis of BL. Inhibition of miR-378a-3p reduced growth of BL cells, confirming its significance in BL. Identification of BL specific target genes of miR-378a-3p revealed four candidates. For two of them, MNT and IRAK4, miR-378a-dependent regulation was confirmed at the protein level. Overexpression of MNT and IRAK4 in BL cell lines resulted in a similar effect as observed upon miR-378a-3p inhibition, suggesting their involvement in the growth regulatory role of miR-378a-3p.

**Abstract:**

MicroRNAs (miRNAs) are small RNA molecules with important gene regulatory roles in normal and pathophysiological cellular processes. Burkitt lymphoma (BL) is an MYC-driven lymphoma of germinal center B (GC-B) cell origin. To gain further knowledge on the role of miRNAs in the pathogenesis of BL, we performed small RNA sequencing in BL cell lines and normal GC-B cells. This revealed 26 miRNAs with significantly different expression levels. For five miRNAs, the differential expression pattern was confirmed in primary BL tissues compared to GC-B cells. MiR-378a-3p was upregulated in BL, and its inhibition reduced the growth of multiple BL cell lines. RNA immunoprecipitation of Argonaute 2 followed by microarray analysis (Ago2-RIP-Chip) upon inhibition and ectopic overexpression of miR-378a-3p revealed 63 and 20 putative miR-378a-3p targets, respectively. Effective targeting by miR-378a-3p was confirmed by luciferase reporter assays for MAX Network Transcriptional Repressor (MNT), Forkhead Box P1 (FOXP1), Interleukin 1 Receptor Associated Kinase 4 (IRAK4), and lncRNA Just Proximal To XIST (JPX), and by Western blot for IRAK4 and MNT. Overexpression of IRAK4 and MNT phenocopied the effect of miR-378a-3p inhibition. In summary, we identified miR-378a-3p as a miRNA with an oncogenic role in BL and identified IRAK4 and MNT as miR-378a-3p target genes that are involved in its growth regulatory role.

## 1. Introduction

Burkitt lymphoma (BL) is one of the fastest growing human tumors with a cell doubling time of about 24 h. BL mainly affects children and young adults but can also occur at a later age [1]. The tumor cells are derived from germinal center B (GC-B) cells and usually carry the hallmark translocation involving *MYC* and the immunoglobulin heavy or light chain loci which results in high expression of MYC [2,3].

MicroRNAs (miRNAs) are a class of short noncoding RNAs of about 22 nucleotides. They modulate gene expression at the post-transcriptional level by translational inhibition or by inducing mRNA degradation [4,5]. MiRNAs regulate a wide range of cellular processes, including cell cycle, proliferation, and apoptosis, and they are important determinants of B-cell development and maturation [6]. A widespread deregulation of miRNA expression has been observed in all B-cell lymphoma subtypes [7].

We and others identified distinct miRNA expression patterns in BL and demonstrated the central role of MYC in regulating miRNA levels [8,9,10,11,12]. Functional studies showed crucial roles for the miR-17~92 cluster, miR-28, miR-150, and miR-155 as either oncogenic or tumor suppressor miRNAs in the pathogenesis of BL [9,13,14,15,16,17,18,19]. Nevertheless, the role of most of the deregulated miRNAs in BL remains to be explored.

In this study, we carried out small RNA-sequencing in BL cell lines and normal GC-B cells and subsequently focused on downstream functional experiments for miR-378a-3p. We show for the first time that this miRNA is upregulated in BL and confirmed its regulation by MYC. Further analysis indicated that miR-378a-3p is essential for the growth of BL cells. Using a combination of genome-wide target gene identification, luciferase reporter assays, and Western blot upon modulating miR-378a-3p levels, we confirmed targeting of Interleukin 1 Receptor Associated Kinase 4 (IRAK4) and MAX Network Transcriptional Repressor (MNT) by miR-378a-3p. Overexpression of these two genes phenocopied the effect of miR-378a-3p inhibition on growth of BL cells.

## 2. Results

### 2.1. MiRNA Expression Profiling in BL and GC-B Cells

**Figure 1:**
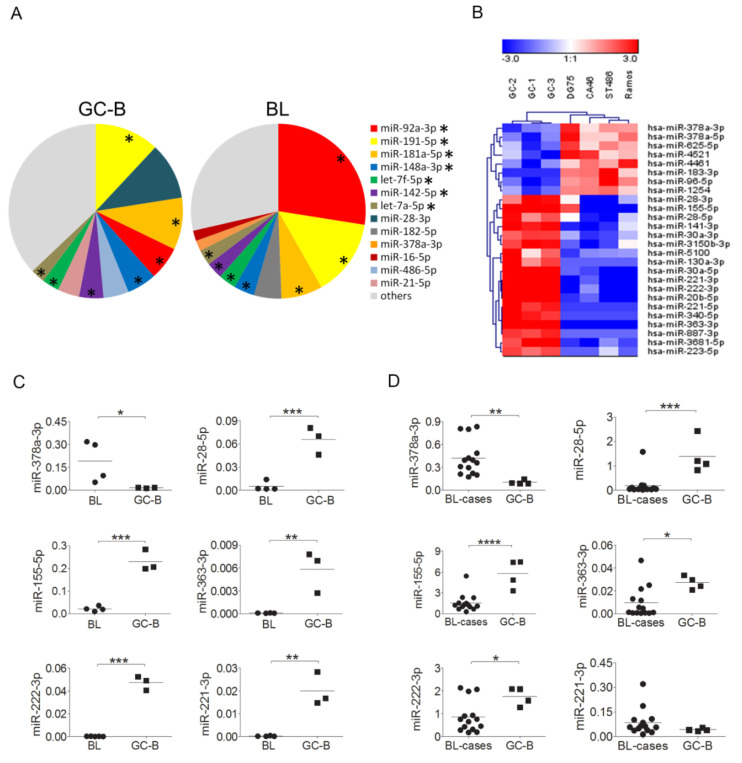
Deregulated expression patterns of microRNAs (miRNAs) in Burkitt lymphoma (BL) compared to germinal center B (GC-B) cells. (**A**) Overview of the top 10 most abundantly expressed miRNAs in Burkitt lymphoma (BL) and normal germinal center B (GC-B) cells as determined by small RNA-sequencing. Asterisks indicate miRNAs present in the top 10 of both BL and GC-B cells. (**B**) Heatmap of miRNAs significantly differentially expressed between BL and GC-B cells. (**C**) qRT-PCR validation results for six of the eight tested miRNAs with significantly differential expression between BL cell lines and GC-B cells. MiRNA expression levels were normalized to Small Nucleolar RNA C/D Box 44 (SNORD44). (**D**) The differential expression pattern was confirmed for five of the six tested miRNAs when BL tissues and GC-B cells were compared. MiRNA expression levels were normalized to Small Nucleolar RNA C/D Box 49 (SNORD49). Significant differences were calculated using an unpaired *t*-test. * *p* < 0.05, ** *p* < 0.01, *** *p* < 0.001, and **** *p* < 0.0001

An overview of the total number of reads and percentages of mapped reads per sample is given in Table S1. The top 10 most abundantly expressed miRNAs accounted for 73% of all reads in BL and for 71% in GC-B cells (total GC-B cells sorted on the basis of a CD20^+^IgD^−^CD38^+^ or IgD^−^CD138^−^CD3^−^CD10^+^ phenotype). Seven of the top 10 most abundantly expressed miRNAs were shared between BL and GC-B cells (Figure 1A). Twenty-six miRNAs were significantly differentially expressed between BL and GC-B cells, including eight miRNAs upregulated in BL and 18 downregulated (Figure 1B). qRT-PCR validation on the same set of samples confirmed differential expression for six out of eight selected miRNAs (Figure 1C and Figure S1). Of the six validated miRNAs, miR-378a-3p levels were increased in BL relative to GC-B cells, while expression levels of miR-28-5p, miR-155-5p, miR-363-3p, miR-221-3p, and miR-222-3p were decreased. Further expression analysis in primary BL tissue samples and GC-B cells confirmed the differential expression for five of the six miRNAs, excluding miR-221-3p (Figure 1D).

### 2.2. MYC-Induced miR-378a-3p Controls BL Cell Growth

**Figure 2:**
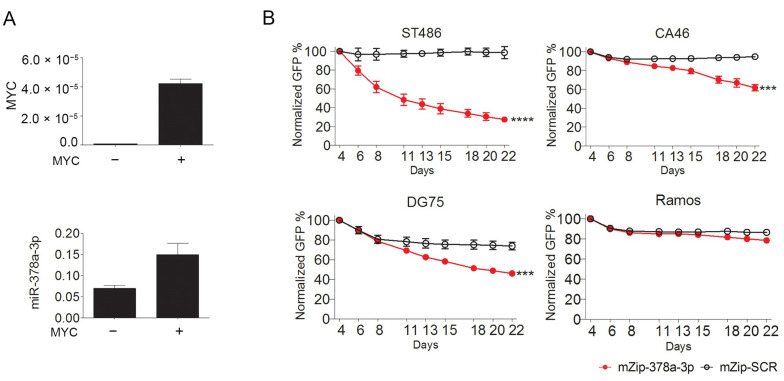
MYC-induced miR-378a-3p is essential for BL cell growth. (**A**) Levels of MYC and miR-378a-3p in tetracycline treated (“−”, MYC-off) and non-treated (“+”, MYC-on) P493-6 B-cells. MYC levels were normalized to RNA Polymerase II Subunit A (POLR2A). MiRNA levels were normalized to SNORD44. (**B**) Green fluorescent protein (GFP) growth competition assay upon miR-378a-3p inhibition in four Burkitt lymphoma (BL) cell lines. The miR-378a inhibitor (mZip-378a-3p) and the scrambled control (mZip-SCR) were stably transduced in BL cells using a lentiviral vector, which co-expresses GFP. The GFP percentage was measured for 18 days, and the GFP percentage at the first day of measurement (day 4) was set to 100%. All assays were performed in triplicate. Significant differences were calculated using a mixed model analysis. *** *p* < 0.001 and **** *p* < 0.0001.

We selected miR-378a-3p for further functional analysis, because it was the only significantly upregulated miRNA with a high expression level in BL. Previous studies demonstrated that miR-378a-3p is induced by MYC in human mammary epithelial cells [20]. We assessed the regulatory role of MYC in B cells using the P493-6 B-cell model that has a tetracycline-repressible MYC allele [21]. Our results showed that this miRNA is also induced by MYC in B cells (Figure 2A).

To explore the role of miR-378a-3p in growth of BL cells, we inhibited miR-378a-3p using a lentiviral miRNA inhibition construct (mZip-378a-3p) in four BL cell lines and followed cell growth in a GFP competition assay. Compared to the negative control (mZip-SCR), a significant decrease in the number of GFP-positive cells was observed in three (ST486, CA46, and DG75) out of four BL cell lines (Figure 2B). No relationship was observed between the reduction in the percentage of GFP^+^ cells and the level of miR-378a-3p expression (Figure S2). Together, our data indicate that inhibition of miR-378a-3p is disadvantageous for BL cells, suggesting miR-378a-3p is indispensable for growth of BL cells.

Overexpression of miR-378a in ST486 using a lentiviral miRNA overexpression construct (pCDH-378a) resulted in a ~47-fold increase in miR-378a-3p level. In a GFP competition assay, miR-378a overexpression had no effects on cell growth, probably due to the already high endogenous levels.

### 2.3. Identification of miR-378a-3p Targets

To identify miR-378a-3p target genes, we performed Ago2-RIP-Chip upon miR-378a-3p inhibition and overexpression in ST486 cells (Figure S3A,B). Efficient pulldown of Ago2-containing RISC and miRNAs was confirmed by qRT-PCR for miR-378a-3p and the unrelated highly expressed miR-181a-5p (Figure S3C,D) and by Western blot for Ago2 protein (Figures S3E and S4). The number of Ago2 immunoprecipitation (IP)-enriched probes was similar in all four conditions, ranging between 6.3% and 9.8% of the probes with consistent expression levels (Table 1).

**Figure 3:**
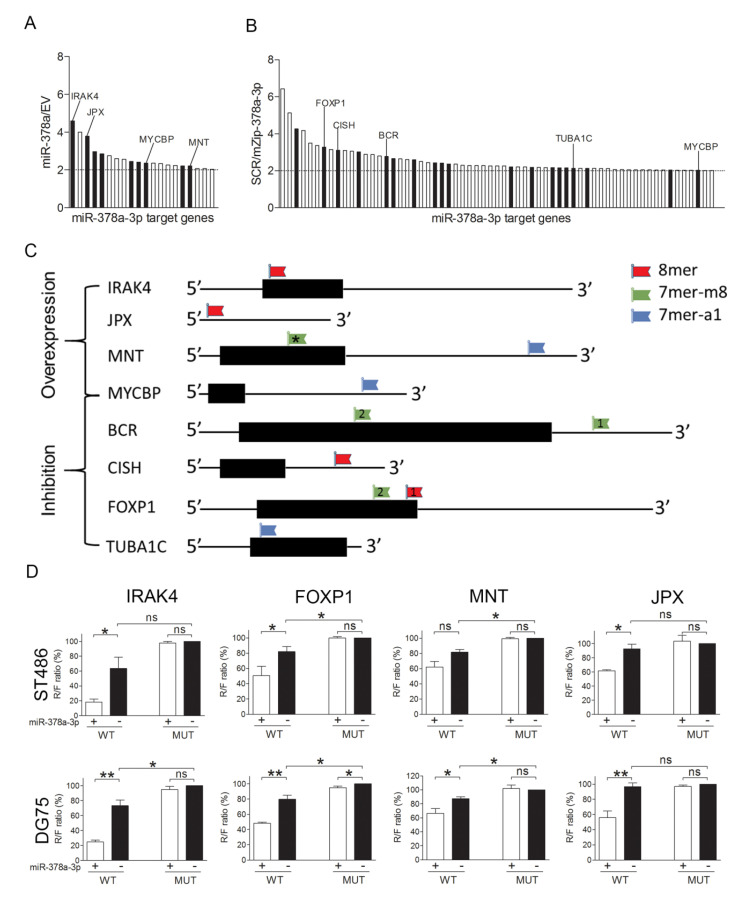
Identification and validation of miR-378a-3p targets. MiR-378a-3p targets identified by Ago2-RIP-Chip upon (**A**) miR-378a overexpression relative to empty vector (EV) and (**B**) scrambled vector relative to miR-378a-3p inhibition (SCR/mZip-378a). The black bars indicate genes with miR-378a-3p seed binding sites. (**C**) Schematic representations of the eight genes selected for luciferase reporter assay validation. Black boxes indicate positions of the open reading frames (ORFs). Positions and types of miR-378a-3p binding sites are indicated relative to the ORF. The binding site in MNT indicated by an asterisk was not tested. (**D**) Luciferase reporter assay results upon co-transfection of ST486 and DG75 cells with the Psi-check-2 construct containing the wildtype (WT) or mutated (MUT) miR-378a-3p binding sites from the selected genes and either an miR-378a-3p mimic or a negative control mimic. Significant differences were calculated using a paired *t*-test. * *p* < 0.05, ** *p* < 0.01, ns = not significant.

A total of 22 probes corresponding to 20 genes showed a ≥2-fold increased IP enrichment upon miR-378a overexpression compared to empty vector control infected cells (Figure 3A and Table 2). Nine of the 20 genes (45%) had at least one putative miR-378a-3p binding site (7mer-A1, 7mer-m8, or/and 8mer). Six of them, i.e., MAX Network Transcriptional Repressor (MNT), Heat Shock Protein Family B (Small) Member 1 (HSPB1), Interleukin 1 Receptor Associated Kinase 4 (IRAK4), Cyclin K (CCNK), Cyclin Dependent Kinase Inhibitor 2A (CDKN2A), and Ring Finger Protein 34 (RNF34), had a Gene Ontology (GO) term related to cell growth, apoptosis, and/or cell cycle. Upon miR-378a-3p inhibition, 74 probes, corresponding to 63 genes, showed a ≥2-fold decreased IP enrichment compared to the negative control infected cells (Figure 3B and Table 3). Nineteen of these 63 genes (30%) contained at least one putative miR-378a-3p binding site, including Cytokine Inducible SH2 Containing Protein (CISH), BCR Activator Of RhoGEF And GTPase (BCR), Tubulin Alpha 1c (TUBA1C), SWI/SNF Related, Matrix Associated, Actin Dependent Regulator Of Chromatin, Subfamily A, Member 4 (SMARCA4), and Forkhead Box P1 (FOXP1) with a GO term related to cell growth, apoptosis, or cell cycle. One of the target genes, i.e., MYC Binding Protein (MYCBP), was identified with both experimental set-ups.

### 2.4. IRAK4 and MNT Are Involved in the Function of miR-378a-3p

For further analysis, we selected MYCBP that was identified in both approaches and six candidates that had at least one 7mer-A1, 7mer-m8, or an 8mer and a GO term related to cell growth, apoptosis, or/and cell cycle (CISH, BCR, TUBA1C, FOXP1, MNT, and IRAK4). We also included the lncRNA JPX, as it showed a strong enrichment upon miR-378a-3p overexpression and contained an 8-mer seed binding site (Figure 3C).

To confirm targeting of the eight selected genes by miR-378a-3p, we carried out luciferase reporter assays for 10 putative miR-378a-3p binding sites in ST486 and DG75. This revealed a strong reduction in the *Renilla*/firefly ratio for four of the miR-378a-3p binding sites in four genes (IRAK4, FOXP1 (site 1), MNT, and JPX) (Figure S5). To further confirm specific binding by miR-378a-3p, we generated constructs with mutations in these four miR-378a-3p binding sites. For IRAK4 (trend), FOXP1, and MNT (both significant), wildtype binding sites showed lower *Renilla*/firefly ratios compared to the mutated binding sites (black bars in Figure 3D), indicating binding of endogenous miR-378a-3p to these sequences. Upon miR-378a-3p overexpression a significantly reduced *Renilla*/firefly ratio was observed for the wildtype, but not for the mutated target sites, confirming efficient and specific targeting (Figure 3D).

**Figure 4:**
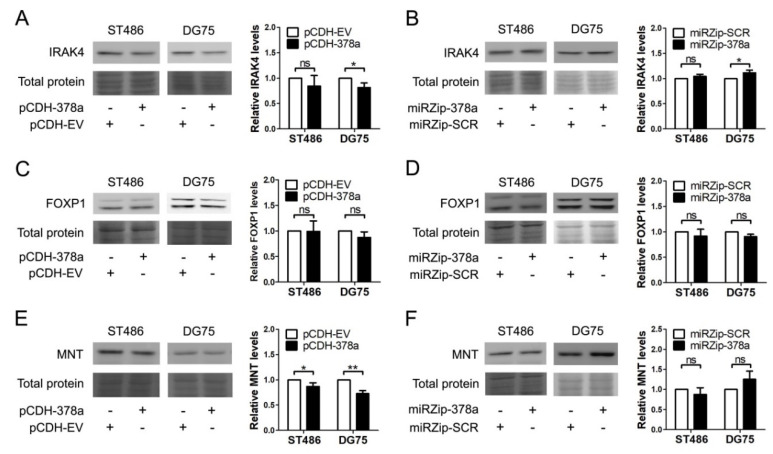
Analysis of the effect of modulating miR-378a-3p levels upon the protein levels of the target genes. Representative examples of the effect of miR-378a overexpression on IRAK4 (**A**), FOXP1 (**C**), and MNT (**E**). Representative examples of the effect of miR-378a-3p inhibition on IRAK4 (**B**), FOXP1 (**D**), and MNT (**F**). cells. Graphs show the quantification of the protein levels of three to four independent infections. For each protein, the part of the total protein lane corresponding to the position of the protein band is shown as an indication for protein loading. Protein levels were quantified relative to the total protein amount as measured in the complete lane. Uncropped blots can be found in Figure S6. Significant differences were calculated using a paired *t*-test. * *p* < 0.05, ** *p* < 0.01, ns = not significant.

To validate the regulatory role of miR-378a-3p on endogenous IRAK4, FOXP1, and MNT protein levels, we analyzed protein levels upon modulation of miR-378a-3p levels in ST486 and DG75 cells (Figure 4 and Figure S6). For IRAK4, this revealed a significant decrease upon miR-378a-3p overexpression and a significant increase upon miR-378a-3p inhibition in DG75. In ST486, the same pattern was observed albeit not significant. MNT levels were significantly decreased upon miR-378a-3p overexpression in DG75 and ST486. Increased MNT levels upon miR-378a-3p inhibition were observed only in DG75 cells although the increase was not significant. No significant differences in FOXP1 expression were observed in either cell line.

**Figure 5:**
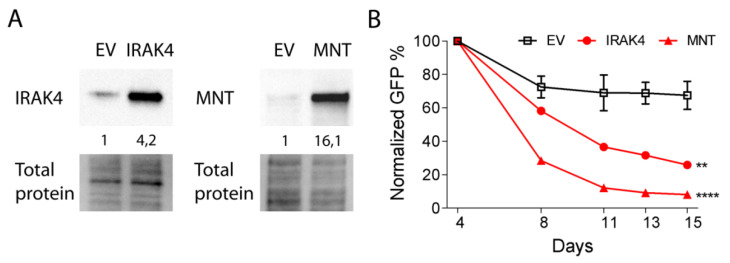
Overexpression of IRAK4 and MNT phenocopies the effect of miR-378a-3p inhibition in DG75 cells. (**A**) Validation of the IRAK4 and MNT overexpression in DG75. An empty vector (EV) was used as a control. For each protein, the part corresponding to the position of the protein band is shown as control of protein loading. Protein levels were quantified relative to the total protein amount in the complete lanes. Uncropped blots can be found in Figure S7. (**B**) GFP competition assays upon overexpression of IRAK4 and MNT resulted in a strong decrease in GFP^+^ cells over time, while no or only a mild effect was observed for the EV control. The GFP percentages were measured for 11 days, and the GFP percentage as measured on day 4 was set to 100%. Assays were performed in duplicate. Significant differences between IRAK4 and MNT overexpression and EV control were calculated using a mixed model analysis. ** *p* < 0.01 and **** *p* < 0.0001.

Next, we performed gain-of-function experiments to determine whether overexpression of the validated targets IRAK4 and MNT could phenocopy the effect of miR-378a-3p inhibition. Overexpression of IRAK4 in DG75 cells resulted in a 74% decrease in GFP^+^ cells in 11 days (Figure 5). Overexpression of MNT resulted in a >90% decrease in GFP^+^ cells in 11 days in DG75. Thus, our results indicate that overexpression of IRAK4 and MNT could phenocopy the effect of miR-378a-3p inhibition on growth of BL cells. Altogether, our results suggest that growth of BL cells depends on high miR-378a-3p levels through regulating IRAK4 and MNT.

## 3. Discussion

In this study, we identified 26 miRNAs differentially expressed in BL cell lines compared to GC-B cells. For five of the miRNAs, deregulated expression levels were confirmed in both BL cell lines and primary tissues. Among them, miR-378a-3p is MYC-induced, highly abundant (top 10 within BL), and overexpressed in BL compared to GC-B cells. Inhibition of miR-378a-3p showed a negative effect on BL cell growth. In a genome-wide Ago2-RIP-Chip analysis, 20 and 63 genes were identified as the potential targets of miR-378a-3p upon miR-378a-3p overexpression and inhibition, respectively. MNT and IRAK4 were confirmed as novel targets of miR-378a-3p in BL, and their overexpression phenocopied the effect of miR-378a-3p on BL cell growth.

Six of the 26 identified differentially expressed miRNAs were reported to be deregulated in BL by Oduor et al. in a previous study [8]. These six included miR-155-5p, miR-221-3p, miR-222-3p, and miR-28-5p which we validated by qRT-PCR in BL cell lines and tissue samples. Nine additional miRNAs were proven to be differentially expressed in BL compared to other B-cell lymphomas [9,10,11,12,20]. Thus, our small RNA sequencing data confirmed some of the previously identified deregulated miRNAs in BL. Moreover, we showed for the first time that miR-378a-3p is an MYC-induced and significantly upregulated miRNA in BL. Since the role of miR-378a-3p in BL was not studied before, we focused on this miRNA for further functional analysis.

Inhibition of miR-378a-3p resulted in a strong reduction of BL cell growth, suggesting a possible oncogenic role of miR-378a-3p in BL. The effect on growth upon miR-378a-3p was most pronounced in ST486. DG75 and CA46 showed intermediate phenotypes while no significant effect was observed in Ramos. There was no obvious relationship between endogenous miR-378a-3p levels and the decrease in percentage of GFP^+^ cells upon miR-378a-3p inhibition. Given the complex interactions between miRNAs and target genes, i.e., multiple targets per miRNA and multiple miRNAs per target, the differences in the observed phenotypes might be related to endogenous levels of other miRNAs or target genes. Previous studies have shown opposite roles of miR-378a-3p in different cancer types. MiR-378a-3p was reported to inhibit growth or promote apoptosis and, thus, act as a tumor suppressor in colorectal cancer, lung cancer, ovarian cancer, prostate cancer, and rhabdomyosarcoma [22,23,24,25,26]. In contrast, in line with our findings, miR-378a-3p was shown to promote proliferation and reduce apoptosis in gastric cancer, nasopharyngeal carcinoma, colorectal cancer, and acute myeloid leukemia [27,28,29,30].

Using an unbiased genome-wide experimental approach, we identified 83 putative miR-378a-3p target genes. Luciferase reporter assays confirmed targeting of four out of eight selected genes, i.e., the protein-coding genes MNT, IRAK4, and FOXP1, and the lncRNA JPX. Targeting of the endogenous *MNT* and *IRAK4* transcripts by miR-378a-3p was confirmed at the protein level, albeit with limited effects. For FOXP1, we could not confirm targeting by miR-378a-3p at the protein level, while we did not follow up potential targeting of endogenous JPX transcripts in BL. Moreover, we showed that overexpression of MNT and IRAK4 strongly inhibited the growth of DG75 cells. Together, these data suggest that the effect of miR-378a-3p might at least in part be dependent on targeting MNT and IRAK4. The apparent discrepancy between the limited effect of miR-378a-3p on the levels of these proteins and the relatively strong phenotype on growth is in line with the general thought that miRNAs most often do not work as on/off switches but rather fine-tune the expression of their targets [5]. A combined moderate effect on MNT, IRAK4, and possible others might explain the strong effect on cell growth. Further experiments in additional cell lines using, e.g., phenotype rescue approaches with more precise control of the overexpression levels are required to confirm the relevance of these targets for the miR-378a-3p-induced phenotype.

Previous studies showed a dual role of MNT in tumorigenesis. On the one hand, MNT was reported as a facilitator of MYC-driven T-cell proliferation and survival [31]. In line with this, a study in Eμ-MYC mice showed that reduced MNT levels reduced tumorigenesis [32], suggesting that MNT is indispensable for MYC-driven oncogenesis. However, in most studies, MNT acted as a tumor suppressor and was a functional antagonist of MYC by repressing its activities related to cell cycle, proliferation, and apoptosis [33,34]. Loss of MNT in mouse embryonic fibroblasts (MEFs) phenocopied the effect of MYC overexpression [35,36,37]. These findings are in line with our results and suggest that high levels of miR-378a-3p could promote BL tumorigenesis by reducing MNT levels, thereby enabling MYC to execute its oncogenic effects in BL.

IRAK4 plays an essential role in the Toll-like receptor (TLR) pathway [38], which mediates inflammatory signals in B cells and causes activation of nuclear factor kappa-light-chain-enhancer of activated B cells (NF-κB). The TLR pathway is hyperactive in mantle cell lymphoma and diffuse large B-cell lymphoma (DLBCL), and activation of NF-κB promotes B-cell survival and proliferation [39,40,41]. Depletion of IRAK4 showed a negative effect on NF-κB activity and autocrine IL-6/IL-10 engagement of the Janus Kinase (JAK)-Signal Transducer and Activator of Transcription 3 (STAT3) pathway, reducing survival of DLBCL cells [42,43]. Despite the pro-survival role of NF-κB in DLBCL, activation of NF-κB has been reported to be disadvantageous in MYC positive BL consistent with our data, supporting a role of miR-378a-3p-dependent repression of IRAK4 in limiting the activation of NF-κB [44,45].

Although we could not show that FOXP1 protein levels are affected by miR-378a-3p, we cannot exclude that expression changes in FOXP1 are more subtle and not captured by our experimental set-up. FOXP1 is a member of the forkhead box (Fox) transcription factor family and a regulator of early B-cell development [46]. Interestingly FOXP1 expression is downregulated during the normal GC reaction. In BL, FOXP1 levels are comparable to GC-B cells, but lower than the level in other B-cell lymphomas [47,48]. The potential relevance of maintaining low FOXP1 in BL is further supported by the finding that aberrant expression of FOXP1 cooperates with (constitutive) NF-κB activity [49], which might be disadvantageous for the survival of BL. The relevance of the long noncoding RNA JPX in the phenotype induced by miR-378a-3p inhibition remains to be elucidated. JPX is an activator of X Inactive Specific Transcript (XIST) and acts as a molecular switch for X chromosome inactivation [50]. JPX was reported to act as an oncogene in ovarian cancer and non-small-cell lung cancer by promoting cell proliferation, invasion, and migration [51,52]. In hepatocellular carcinoma, JPX-dependent induction of XIST suppresses hepatocellular carcinoma progression by binding to the miR-155-5p oncomiR [53]. The potential role of miR-378a-3p in regulating FOXP1 and JPX levels need to be further established.

## 4. Materials and Methods

### 4.1. BL Cell Lines, Germinal Center (GC) B Cells, and BL Patient Material

BL cell lines were purchased from American Type Culture Collection (ATCC/LGC Standards, Molsheim Cedex, France) (ST486 and Ramos) and German Collection of Microorganisms and Cell Cultures (DSMZ, Braunschweig, Germany) (CA46 and DG75). BL cells were cultured at 37 °C under an atmosphere containing 5% CO_2_ in Roswell Park Memorial Institute (RPMI)-1640 medium (Cambrex Biosciences, Walkersville, MD, USA) supplemented with 2 nM ultra-glutamine, 100 U/mL penicillin, 0.1 mg/mL streptomycin, and 10% (CA46, DG75, and Ramos) or 20% (ST486) fetal bovine serum (Sigma-Aldrich, Zwijndrecht, The Netherlands). P493-6 B-cells were cultured as described previously [54]. We routinely confirmed cell line identity using the PowerPlex^®^ 16HS System (Promega, Leiden, The Netherlands) and absence of mycoplasma contamination.

GC-B cells and frozen BL tissue sections were obtained previously as described in [9,54,55]. GC-B cells (defined as CD20^+^IgD^−^CD38^+^, *n* = 6 or IgD^−^CD138^−^CD3^−^CD10^+^, *n* = 1) were sorted from routinely removed tonsil specimens of children. Specifically, for small RNA seq experiments, fluorescence-activated cell sorter (FACS)-sorted (CD20^+^IgD^−^CD38^+^, *n* = 2) and magnetic-activated cell sorter MACS-sorted (IgD^−^CD138^−^CD3^−^CD10^+^, *n* = 1) GC-B cells were used as controls, while FACS-sorted (CD20^+^IgD^−^CD38^+^, *n* = 4) GC-B cells were used for qRT-PCR validation experiments. Written permission for the use of the tonsil tissues to isolate GC-B cells was obtained from the parents of the children. The study protocol was consistent with international ethical guidelines (the Declaration of Helsinki and the International Conference on Harmonization Guidelines for Good Clinical Practice). According to the Medical ethics review board of the University Medical Center Groningen our studies fulfilled requirements for patient anonymity and were in accordance with their regulations. The Medical ethics review board waives the need for approval if rest material is used under law in the Netherlands, and waives the need for informed consent when patient anonymity is assured (BL tissue samples).

### 4.2. RNA Isolation

RNA was isolated using miRNeasy Mini or Micro kit (Qiagen, Hiden, Germany) according to the manufacturer’s instructions. RNA concentration was measured by a NanoDropTM 1000 Spectrophotometer (Thermo Fisher Scientific Inc., Waltham, MA, USA) and integrity was evaluated on a 1% agarose gel.

### 4.3. Small RNA Library Preparation and Sequencing

Firstly, 1–2 µg of total RNA from four BL cell lines and three samples of GC-B cells was used to generate small RNA libraries using Truseq Small RNA Sample Preparation Kit and TruSeq small RNA indices (Illumina, San Diego, CA, USA). Sequencing was performed on an Illumina 2000 HiSeq platform. After removal of 3′- and 5′-adaptor sequences from the raw reads using the CLC Genomics Workbench (CLC Bio, Cambridge, MA, USA), sequencing data were analyzed using miRDeep version 2.0 (Max Delbrück Center for Molecular Medicine in the Helmholtz Association, https://www.mdc-berlin.de/8551903/en) [56] and annotated against miRbase version 21 (http://www.mirbase.org) [57] allowing one nucleotide mismatch. Read counts of miRNAs with the same mature miRNA sequence were merged. Total read counts per sample were normalized to 1,000,000. For statistical analysis, we included all unique miRNAs with at least 50 read counts in all seven samples. Genesis software v1.7.6 (Institute for Genomics and Bioinformatic Graz, Graz, Austria) was used to generate the heat map. The small RNA sequencing data were deposited in the Gene Expression Omnibus database (http://www.ncbi.nlm.nih.gov/geo; accession number GSE92616).

### 4.4. qRT-PCR

For validation of small RNA sequencing results, we selected differentially expressed miRNAs with expression log_2_ reads per million (RPM) >8 in BL or GC-B cells. We selected the most optimal Taqman assay on the basis of isoform abundance as observed in the small RNA sequencing data (Table S2). The miRNA expression levels were analyzed using Taqman miRNA quantitative PCR assays (Thermo Fisher Scientific Inc.) in a multiplexed fashion as described previously [58]. Cycle crossing point (Cp) values were determined with Light Cycler 480 software version 1.5.0 (Roche, Basel, Switzerland). Relative expression levels of miRNAs to the housekeeping gene (SNORD44 or SNORD49) were determined by calculating 2^−ΔCp^ (ΔCp = Cp_miRNA_ − Cp_house-keeping gene_). MYC transcript levels were analyzed as indicated previously [54].

### 4.5. Lentiviral Constructs, Transduction, and GFP Competition Assay

Lentiviral constructs to inhibit (mZip-378a-3p) or overexpress (pCDH-miR-378a) miR-378a-3p were purchased from System Biosciences (Palo Alto, CA, USA). A nontargeting mZip-scrambled (SCR) and an empty vector pCDH (EV) construct were used as negative controls. Lentiviral pLV-EGFP:T2A:Puro-EF1A IRAK4, MNT, and empty vector control constructs were purchased from VectorBuilder (Chicago, IL, USA). Lentiviral particles were produced in HEK-293T cells by calcium phosphate precipitation transfection using a third-generation packaging system as described previously [55]. Briefly, HEK-293T cells were seeded in six-well plates and grown until ~80% confluence. A plasmid mix consisting of 15 μL of CaCl_2_ (2.5 M), 1 μg of pMSCV-VSV-G, 1 μg of pRSV.REV, 1 μg of pMDL-gPRRE, 2 μg of lentiviral vector, and 150 μL of 2× HEPES buffered saline (HBS) was prepared to transfect the HEK-293T cells. The virus was harvested and filtered using a 0.45 μm filter 48 h after transfection. The virus was either used directly or stored at −80 °C.

For GFP competition assays, BL cell lines were infected with the mZip-378a-3p and the negative control mZip-SCR in three biological replicates per construct, aiming at an infection efficiency of 20% to 50% GFP^+^ cells on day 4. The percentage of GFP^+^ cells was monitored by flow cytometry (BD Biosciences, San Jose, CA, USA) three times per week for a total period of 18 days. For the IRAK4 and MNT GFP competition assays, two biological replicates per construct were performed, and the percentage of GFP^+^ cells was monitored for 11 days.

### 4.6. AGO2-IP Procedure

Immunoprecipitation (IP) of the Ago2-containing RNA-induced silencing complex (RISC) (Ago2-IP) procedure was done as described previously [59]. To identify miR-378a-3p target genes, we applied the Ago2-RIP-Chip on BL cells infected with lentiviral miR-378a-3p inhibition or overexpression constructs. For both constructs, a parallel infection with appropriate control constructs (nontargeting or empty vector) was performed. We aimed at a high infection percentage and harvested the cells at day 5, either directly or after sorting to reach a GFP^+^ percentage >95% for inhibition and >85% for overexpression. For each AGO2-IP experiment, we started with ~30 million cells. RNA was isolated from the Ago2-IP and total (T) fractions. Efficiency of the Ago2-IP procedure was confirmed by qRT-PCR for miR-378a-3p and miR-181a and by Western blot for the Ago2 protein.

### 4.7. Western Blotting

Infected cells were harvested and lysed in lysis buffer (#9803, Cell Signaling Technology, Danvers, MA, USA) supplemented with protease inhibitor. After centrifugation at 14,000 rpm for 10 min (4 °C), supernatants were collected, and protein concentrations were measured using the bicinchoninic acid (BCA) Protein Assay Kit (Thermo Fisher Scientific Inc., Waltham, MA, USA) according to the manufacturer’s protocol. Then, 20 µg of protein was separated on a polyacrylamide gel and transferred to a nitrocellulose membrane followed by incubation overnight at 4 °C with primary antibodies, anti-Ago2 (2E12-1C9, Abnova, Taipei, Taiwan), anti-MNT (#A303-626A, Bethyl Lab, Montgomery, TX, USA), anti-IRAK4 (ab32511, Cambridge, UK), and anti-FOXP1 (#2005, Cell Signaling Technology, Danvers, MA, USA)). All antibodies were diluted 1000× in 5% milk in Tris-buffered saline with Tween-20 (TBST). After incubation with the secondary and tertiary (for MNT, IRAK4, and FOXP1) antibodies and with the enhanced chemiluminescence (ECL) substrate (Thermo Fisher Scientific Inc.), chemiluminescence was detected. Ago2 was quantified with Image Lab 4.0.1 software (BioRad, Hercules, CA, USA), and MNT, IRAK4, and FOXP1 were quantified using ImageJ (NIH, Bethesda, MD, USA). MNT, IRAK4, and FOXP1 protein levels were normalized relative to the total protein amount in the complete lane.

### 4.8. Microarray Analysis

About 50 ng RNA of both the total (T) and the IP fractions were labeled and hybridized on an Agilent gene expression microarray (AMADID no.: 072363, SurePrint G3 Human Gene Exp v3 array kit, Agilent Technologies, Santa Clara, CA, USA). The microarray contained 58,341 probes against coding and noncoding transcripts. The procedure and data analysis were performed as previously described [54]. Briefly, after complementary RNA (cRNA) synthesis and amplification, labeling was done with cyanine 3-CTP (Cy3) or cyanine 5-CTP (Cy5) using the LowInput QuickAmp Labeling kit (catalog no.: 0006322867). Equal amounts of Cy3- or Cy5-labeled cRNA samples were mixed and hybridized on the microarray slide overnight. Raw data were quantile normalized without baseline transformation using GeneSpring GX 12.5 software (Agilent Technologies). Probes were selected for further analysis if they were flagged present in all samples, expressed in the 25th to 100th percentile in at least half of the total (T) fractions, and showed consistent expression in the duplicate measurements (<2-fold change). The average signals of replicates were used to calculate the IP/T ratio, and probes with a ≥ 2-fold enrichment in the IP fraction as compared to total (T) fraction were considered as potential miRNA targets.

For pCDH-378a transduced cells, we next assessed miR-378a-3p targets by identifying probes that were enriched at least ≥2-fold higher in miR-378a-overexpressing cells as compared to empty vector (pCDH-EV). For mZip-378a-3p transduced cells, we assessed miR-378a-3p targets by identifying probes with at least ≥2-fold higher enrichment in mZip-SCR transduced cells as compared to mZip-378a-3p cells. Gene expression microarray data are deposited in the Gene Expression Omnibus database (http://www.ncbi.nlm.nih.gov/geo; accession number: GSE141691).

### 4.9. Identification of miR-378a-3p Seed Sites and Gene Ontology (GO) Terms

For all Ago2-RIP-Chip identified targets of miR-378a-3p, we used a Perl script to search for 7mer-A1, 7mer-m8, and 8mer [60] miR-378a-3p seed sites in 5′-untranslated region (UTR), coding sequence (CDS), and 3′-UTR). Ensemble transcript isoforms were selected on the basis of a refseq identifier (ID) conversion using Biomart (https://www.ensembl.org/biomart). For lncRNA transcripts without an Ensembl ID, we used the LNCipedia or a XLOC/TCONS (BROAD Institute, Cambridge, MA, USA) transcript ID. The growth-related Gene Ontology (GO) terms (proliferation, cell cycle, and apoptosis) for selected genes were retrieved from the Ensembl database (https://www.ensembl.org/biomart).

### 4.10. Cloning of miRNA Binding Sites and Luciferase Reporter Assay

We adapted the psi-Check-2 vector (Promega, Madison, WI, USA) to remove the predicted miR-378a-3p target site (7mer-A1) in the open reading frame of the *Renilla* luciferase gene. The binding site was mutated by changing two nucleotides in the seed region without affecting the amino-acid sequence. This was accomplished by substituting the 460 nt long fragment between the EcoRV and XhoI sites of the psi-Check-2 vector (Figure S8; Integrated DNA Technologies, Leuven, Belgium). Effective *Renilla* luciferase production independent of miR-378a-3p levels was confirmed before cloning putative binding sites of target genes.

Ten potential miRNA binding sites of eight miR-378a-3p target genes were ordered as 58-mer oligo duplexes (Integrated DNA technologies) and cloned into the *Xho*I and *Not*I restriction sites of the modified luciferase reporter vector (Table S3). For the binding sites with a positive result in the first luciferase reporter assay, mutant controls were generated by cloning oligo duplexes with mutations in three nucleotides in the seed region. The reporter vectors with miR-378a-3p wildtype or mutated binding sites were co-transfected with 10 µM of either miR-378a pre-miRNA (Cat. NO.: AM17100, Ambion, Austin, TX, USA) or control oligos (Cat. NO.: AM17111, Ambion) to ST486 and DG75 cells using an Amaxa nucleofector device (program A23) and the Amaxa Cell Line Nucleofector Kit V (Cat NO.: VACA-1003) (Amaxa, Gaithersburg, MD, USA). Cells were harvested 24 h after transfection. *Renilla* and firefly luciferase activity in cell lysates was measured using a Dual-Luciferase Reporter Assay System (Promega). Each experiment was measured in duplicate and results were averaged per experiment. For each construct, the luciferase assay was performed in three independent biological replicates.

### 4.11. Statistical Analysis

MiRNAs significantly differentially expressed in the small RNA-seq profiling were identified with a moderated *t*-test and Benjamini–Hochberg correction for multiple testing using the GeneSpring GX software (version 12.5, Agilent Technologies, Santa Clara, CA, USA). For confirmation of differentially expressed miRNAs by qRT-PCR, we used the nonparametric Mann–Whitney U-test (GraphPad Software Inc., San Diego, CA, USA). Statistical analysis of GFP competition assays was performed as described previously [55]. Briefly, the percentage of mZip-378a-3p infected cells at day 4 was set to 100% and compared to percentages in the control over time using a mixed model, with time and the interaction of time and miRNA construct type as a fixed effect and the measurement repeat within miRNA construct type as a random effect in SPSS (22.0.0.0 version, IBM, Armonk, NY, USA). For the luciferase reporter assay, significance was calculated on the basis of the *Renilla*-to-firefly (RL/FL) luciferase ratios between experimental samples and negative controls using a paired *t*-test (GraphPad Software Inc.). MNT, IRAK4, and FOXP1 protein levels were normalized to total protein loading as visualized with Ponceau S staining. The average change in protein levels of three to four experiments was calculated relative to pCDH-EV and miRZip-SCR controls, which were set to 1. Significance was calculated using a one-tailed paired *t*-test (GraphPad Software Inc.).

## 5. Conclusions

In conclusion, we identified 26 miRNAs differentially expressed between BL cells and GC-B cells and confirmed deregulated expression of five out of eight miRNAs in both BL cell lines and tissue samples. For one of the differentially and highly abundant (top 10 in BL) miRNAs, miR-378a-3p, we showed a negative effect on BL cell growth upon inhibition. Overexpression of two experimentally proven miR-378a-3p targets, i.e., IRAK4 and MNT, phenocopied the effect observed upon miR-378a-3p inhibition. Together, our data show a critical role for miR-378a-3p in promoting BL cell growth and suggest that this involves controlling IRAK4 and MNT levels.

## Figures and Tables

**Table 1:** Number of genes in the miRNA targetome upon miR-378a-3p overexpression (pCDH) and inhibition (mZip).

IP/T Ratio	pCDH (*n* = 9233)			mZip (*n* = 8944)		
	EV	378a	378a/EV	SCR	mZip-378a-3p	SCR/mZip-378a-3p
≥2	611	586	20	741	878	63
≥4	117	117	2	171	196	4
≥8	25	18	0	53	34	0

EV = pCDH-EV, 378a = pCDH-378a, SCR = mZip-SCR, IP = Ago2 immunoprecipitated fraction, T = total fraction.

**Table 2:** Identified targets of miR-378a-3p upon overexpression.

Gene	Transcript ID	IP/T Ratio			miR-378a-3p Binding Site			Growth-Related GO
		EV *	378a	FC	5′UTR	CDS	3′UTR	
*IRAK4*	ENST00000613694	1.0	4.6	4.6		8m		yes
*CDKN2A*	ENST00000304494	1.0	4.0	4.0				yes
*JPX*	ENST00000415215	1.5	5.8	3.9			8m **	
*PLGRKT*	ENST00000223864	1.0	3.0	3.0	8m			
*TMEM245*	ENST00000374586	1.0	2.9	2.9			7m8/8m	
*TOMM6*	ENST00000398884	1.5	4.1	2.7				
*CDK1*	ENST00000395284	1.0	2.6	2.6				yes
*FAM117A*	ENST00000240364	1.4	3.6	2.6				
*WDR83OS*	ENST00000596731	1.3	3.2	2.5	7m8			
*CCNK*	ENST00000389879	1.0	2.4	2.4		8m		yes
*MYCBP*	ENST00000397572	2.3	5.4	2.3			7mA1	
*RNF34*	ENST00000392465	1.3	3.0	2.3				yes
*UBC*	ENST00000339647	1.0	2.3	2.3				yes
*POP4*	ENST00000585603	1.0	2.3	2.3				
*MNT*	ENST00000174618	1.2	2.7	2.3		7m8	7mA1	yes
*HSPB1*	ENST00000248553	1.8	3.9	2.2	7mA1			yes
*INAFM1*	ENST00000552360	1.3	2.8	2.2				
*PCNA*	ENST00000379160	1.0	2.1	2.1				yes
*SP100*	ENST00000264052	1.2	2.5	2.1				
*RPP25L*	ENST00000297613	1.2	2.4	2.0				

* IP/T ratios in pCDH-EV (EV) were set to 1.0 in cases where the ratios were <1.0. ** The binding site in the noncoding RNA is listed in the 3′-UTR column. 7mA1 = 7mer-A1, 7m8 = 7mer-m8, 8m = 8mer, FC = fold change, 378a = pCDH-378a, IP = Ago2 immunoprecipitated fraction, T = Total fraction, 5′UTR = 5′ untranslated region, CDS = coding sequence, 3′UTR = 3′ untranslated region, GO = gene ontology.

**Table 3:** Identified targets of miR-378a-3p upon inhibition.

Gene	Transcript ID	IP/T Ratio			miR-378a-3p Binding Site			Growth-Related GO
		mZip-378a-3p *	SCR	FC	5′UTR	CDS	3′UTR	
*DYNLRB1*	ENST00000357156	1.0	6.4	6.4				
*VPS18*	ENST00000220509	8.5	43.4	5.1				
*NAPA-AS1*	ENST00000594367	1.2	5.2	4.3			7mA1 **	
*C11orf95*	ENST00000433688	1.0	4.2	4.2				
*HOMEZ*	ENST00000357460	1.2	4.3	3.5				
*TMEM79*	ENST00000405535	1.7	5.7	3.4				
*FOXP1*	ENST00000493089	4.5	14.9	3.3		7m8/8m		yes
*PCIF1*	ENST00000372409	1.0	3.2	3.2		7m8		
*ATP6V0C*	ENST00000330398	1.0	3.1	3.1				
*CISH*	ENST00000348721	2.1	6.6	3.1			8m	yes
*lnc-FOXB1-8*	lnc-FOXB1-8:1	1.0	3.3	3.1				
*MT1B*	ENST00000334346	1.1	3.5	3.1				yes
*lnc-EGLN1-1*	lnc-EGLN1-1:6	1.0	2.9	2.9				
*NELFA*	ENST00000382882	1.0	3.0	2.9				
*BCR*	ENST00000305877	1.0	2.8	2.8		7m8	7m8	yes
*MT1L*	ENST00000565768	1.2	3.2	2.8				
*FAT3*	ENST00000409404	1.0	2.7	2.7		8m	7mA1	
*TRAF3IP2-AS1*	ENST00000525151	1.0	2.7	2.7				
*C22orf39*	ENST00000611555	1.0	2.6	2.6				
*KRTCAP2*	ENST00000295682	1.6	4.1	2.6				
*LINC01122*	ENST00000427421	11.2	29	2.6			7mA1 **	
*ACTG1P20*	ENSG00000241547	1.3	3.2	2.5				
*lnc-KRTAP5-10-1*	lnc-KRTAP5-10-1:1	1.5	3.5	2.4			7m8 **	
*MT1E*	ENST00000306061	1.1	2.8	2.4				yes
*NUDT19*	ENST00000397061	1.0	2.5	2.4			7mA1	
*PRDX4*	ENST00000379341	1.0	2.4	2.4				yes
*TMEM258*	ENST00000537328	1.1	2.6	2.4				
*XLOC_l2_005952*	TCONS_l2_00011050	6.6	15.6	2.4		7m8		
*BTG3*	ENST00000629582	1.6	3.7	2.3				yes
*EVI5L*	ENST00000270530	1.3	3.0	2.3				
*LINC01534*	ENST00000433232	1.0	2.3	2.3				
*lnc-ADA-1*	lnc-ADA-1:2	1.0	2.3	2.3				
*lnc-ZNF431-4*	lnc-ZNF431-4:1	1.1	2.6	2.3				
*MT1A*	ENST00000290705	2.5	5.6	2.3				yes
*CSRP2*	ENST00000311083	2.7	5.9	2.2				
*FYCO1*	ENST00000296137	1.0	2.2	2.2			7m8	
*HYAL3*	ENST00000336307	2.0	4.3	2.2				
*KCNQ1*	ENST00000632153	1.0	2.2	2.2				
*lnc-RP11-158I9.5.1-2*	TCONS_00019776	1.5	3.3	2.2				
*PCNX*	ENST00000304743	3.8	8.3	2.2		7mA1/8m		
*PRSS36*	ENST00000268281	1.4	2.8	2.2				
*SMARCA4*	ENST00000344626	1.0	2.2	2.2		7m8		yes
*TNRC6C*	ENST00000335749	8.5	18.3	2.2		7m8		
*TOLLIP*	ENST00000317204	1.3	2.8	2.2			7mA1	
*ARF4*	ENST00000303436	1.0	2.1	2.1				yes
*ATG4D*	ENST00000309469	1.4	2.8	2.1				yes
*CSE1L*	ENST00000262982	1.2	2.5	2.1				yes
*lnc-PCF11-1*	lnc-PCF11-1:12	1.5	3.2	2.1				
*MARS*	ENST00000262027	1.7	3.6	2.1				
*NANS*	ENST00000210444	1.0	2.1	2.1				
*ORMDL2*	ENST00000243045	1.1	2.3	2.1				
*PFKFB2*	ENST00000367080	1.4	3.0	2.1		7m8	7mA1	
*PGM2L1*	ENST00000298198	1.8	3.7	2.1				
*PTPN23*	ENST00000265562	1.5	3.1	2.1				
*TSACC*	ENST00000368255	1.5	3.1	2.1				
*TUBA1C*	ENST00000301072	1.1	2.4	2.1		7mA1		yes
*XLOC_l2_013031*	TCONS_l2_00024809	6.5	13.7	2.1				
*C15orf61*	ENST00000342683	1.3	2.6	2.0			8m	
*LAT*	ENST00000360872	1.0	2.0	2.0				
*LOC101929494*	N/A	1.6	3.2	2.0				
*MYCBP*	ENST00000397572	1.5	3.1	2.0			7mA1	
*NDRG4*	ENST00000394279	1.1	2.2	2.0				yes
*TUBE1*	ENST00000368662	1.1	2.2	2.0				yes

* IP/T ratios in mZip-378a-3p were set to 1.0 if <1.0. ** Binding sites on noncoding RNAs are listed in 3′-UTR column. 7mA1 = 7mer-A1, 7m8 = 7mer-m8, 8m = 8mer, FC = fold change, SCR = mZip-SCR, N/A = not available, IP = Ago2 immunoprecipitated fraction, T = total fraction, 5′UTR = 5′ untranslated region, CDS = coding sequence, 3′UTR = 3′ untranslated region, GO = gene ontology.

## Supplementary Materials

The following are available online at https://www.mdpi.com/2072-6694/12/12/3546/s1, Figure S1: Validation of the differential expression pattern observed in the BL cell lines relative to GC-B cells by qRT-PCR, Figure S2: miR-378a-3p levels based on small RNA-seq data derived from individual BL cell lines and germinal center B-cells, Figure S3: Validation of Ago2-IP procedure on ST486 cells upon inhibition and overexpression of miR-378a-3p, Figure S4: Uncropped western blot results for Ago2 protein, Figure S5: Results of the luciferase reporter assays for 10 putative miR-378a-3p binding sites identified in eight Ago-2 IP-enriched genes in (A) ST486 and (B), Figure S6: Uncropped Western blot results for IRAK4, FOXP1, and MNT upon miR-378a-3p inhibition or overexpression, Figure S7: Uncropped Western blot results for IRAK4 and MNT upon lentiviral overexpression, Figure S8: Sequence of the minigene used to generate a luciferase reporter vector without the putative miR-378a-3p binding site in the *Renilla* gene, Table S1: Small RNA sequencing read summary, Table S2: Taqman miRNA assays used for qRT-PCR validation, Table S3: Oligos for cloning miR-378a-3p binding sites from selected genes, wildtype, and with mutations in miR-378a-3p seed. Green letters indicate miR-378a-3p binding sites and red letters indicate the mismatches.
