# Supplementary material for: MiR-378a-3p Is Critical for Burkitt Lymphoma Cell Growth
Source: Cancers (Basel). 2020 Nov 27;12(12):3546. doi: 10.3390/cancers12123546 (PMC7760147; doi:10.3390/cancers12123546)
Supplement: Supplementary file 1 [file cancers-12-03546-s001.pdf]

cancers

Supplementary Materials: MiR-378a-3p Is Critical for Burkitt Lymphoma Cell Growth

Fubiao Niu, Agnieszka Dzikiewicz-Krawczyk, Jasper Koerts, Debora de Jong, Laura Wijenberg, Margot Fernandez Hernandez, Izabella Slezak-Prochazka, Melanie Winkle, Wierd Kooistra, Tineke van der Sluis, Bea Rutgers, Miente Martijn Terpstra, Klaas Kok, Joost Kluiver and Anke van den Berg

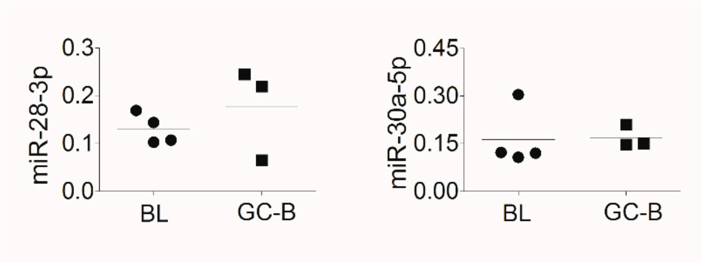

Figure S1. qRT-PCR validation of the differential expression pattern observed by small RNA-seq in the BL cell lines relative to GC-B cells . The decreased levels of miR-28-3p and miR-30a-5p in BL cell lines relative to GC-B cells could not be validated by qRT-PCR. miRNA levels were normalized to RNU44.

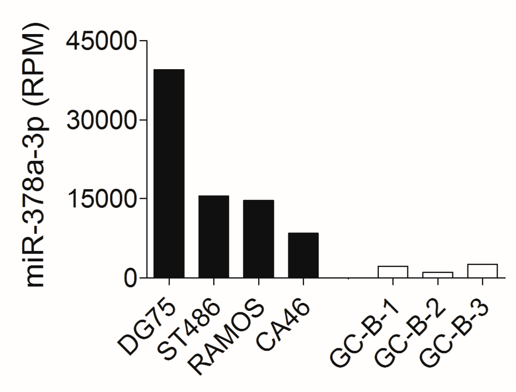

Figure S2. miR-378a-3p levels based on small RNA-seq data derived from individual BL cell lines (black bars) and germinal center B-cells (GC-B, white bars). RPM = reads per million.

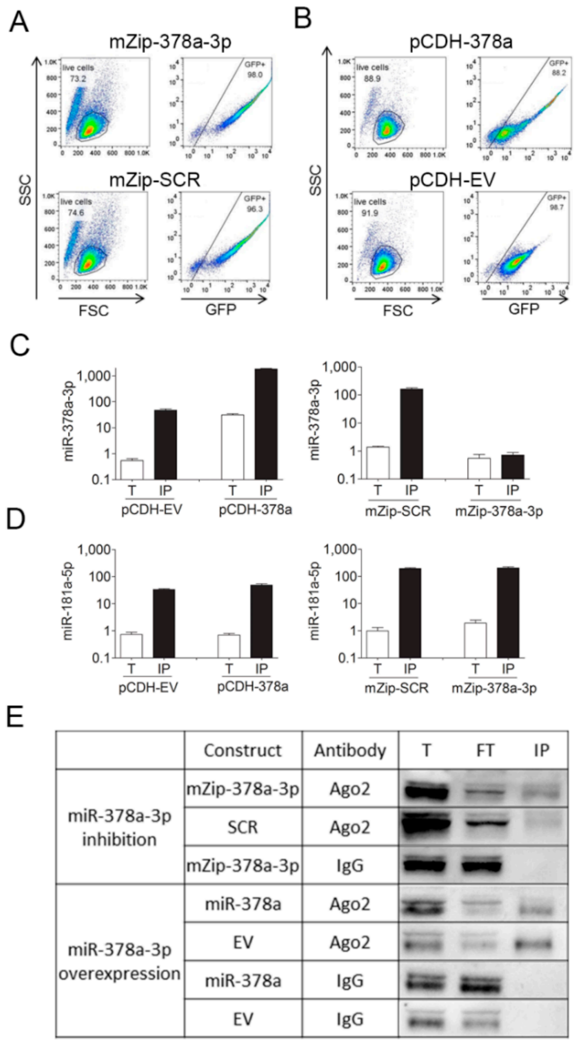

Figure S3. Validation of Ago2-IP procedure in ST486 cells upon inhibition and overexpression of miR-378a-3p. Infection efficiency for (**A**) miR-378a-3p inhibition and (**B**) overexpression constructs in ST486. Enrichment of (**C**) miR-378a-3p and (**D**) a randomly selected control miRNA, miR-181a-5p in the Ago2 immunoprecipitated (IP) fraction in comparison to the total (T) fraction. (**E**) Western blot results of Ago2 protein in T, flow through (FT), and IP samples using the anti-Ago2 antibody or the control anti-IgG antibody for immunoprecipitation.

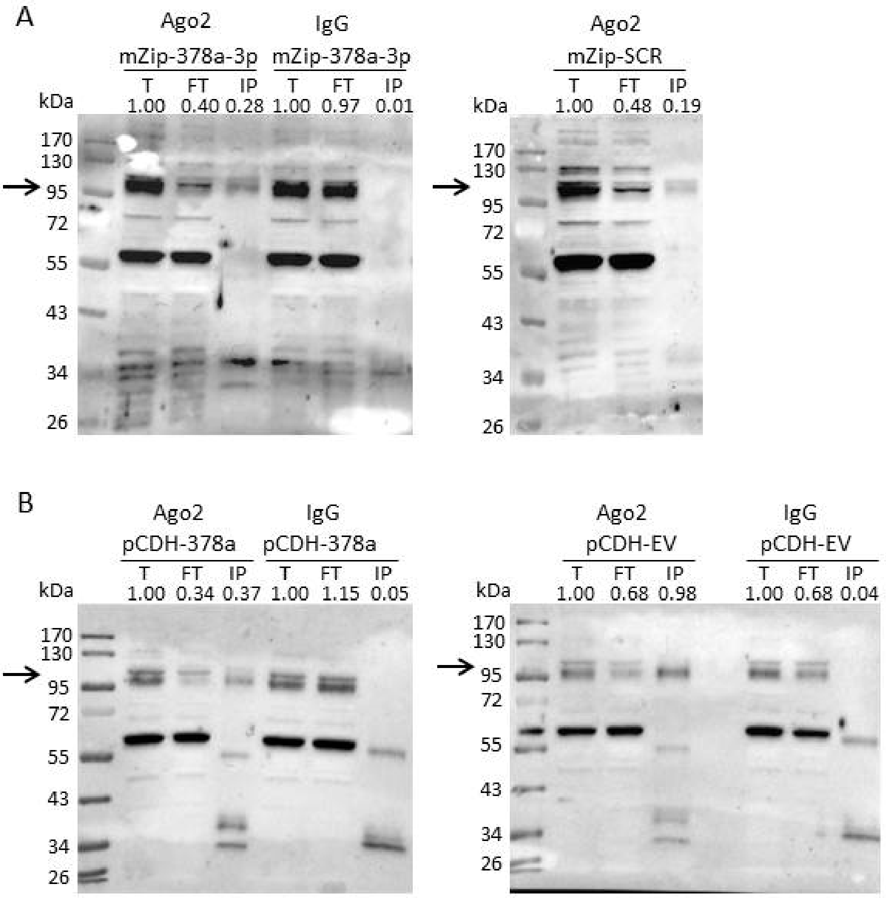

Figure S4. Uncropped western blot results for Ago2 protein in Total fraction (T), flow through fraction (FT), and Ago2-immunoprecipitation fraction (IP) samples using the anti-Ago2 antibody or the control anti-IgG antibody for miR-378a-3p inhibition (**A**) and overexpression (**B**) experiments (see also Figure S3E). Arrows indicate the AGO2 protein. For each IP experiment the total fraction is set to 1.

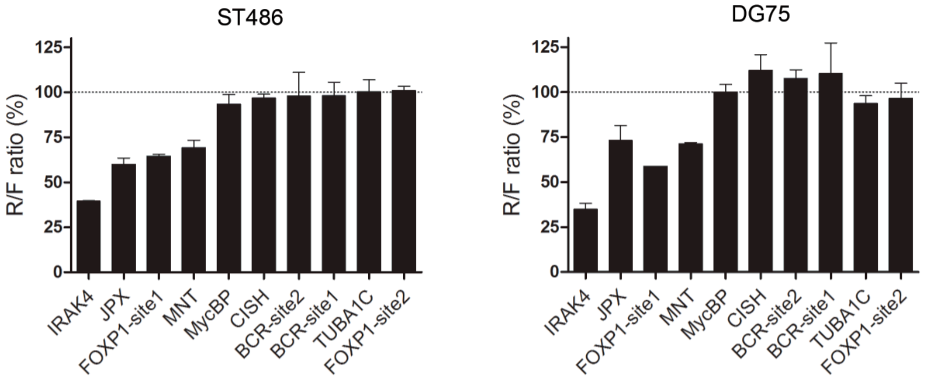

Figure S5. Results of the luciferase reporter assays for 10 putative miR-378a-3p binding sites identified in 8 Ago-2 IP enriched genes in (**A**) ST486 and (**B**) DG75. The 10 binding sites were cloned to the Psi-check-2 vector and co-transfected with miR-378a-3p mimics or negative control mimics to ST486 and DG75 BL cells. Binding of miR-378a-3p was confirmed for IRAK4, JPX, FOXP1-site1 and MNT in both cell lines.

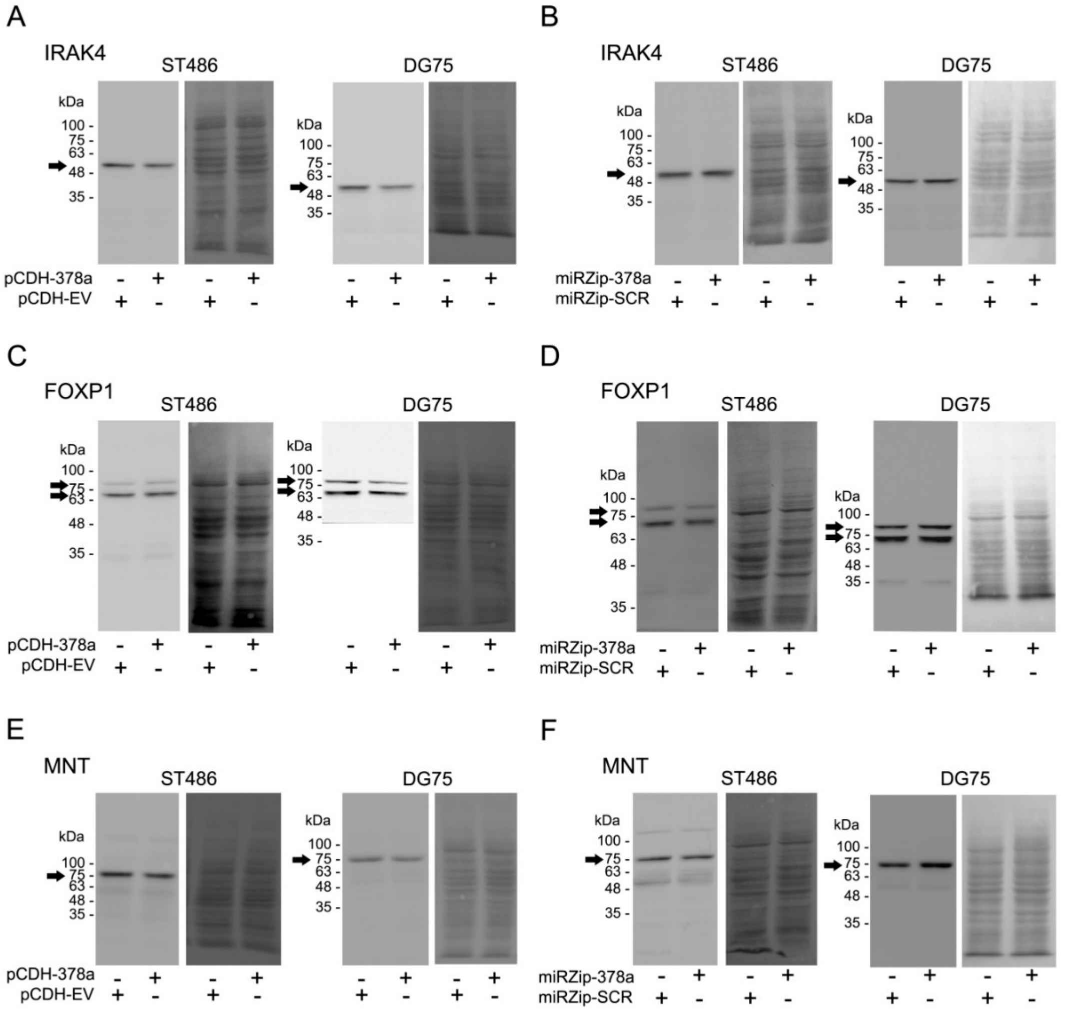

Figure S6. Uncropped western blot results for IRAK4 (A & B), FOXP1 (C & D) and MNT (E & F) as presented in Figure 4. Arrows indicate the protein of interest.

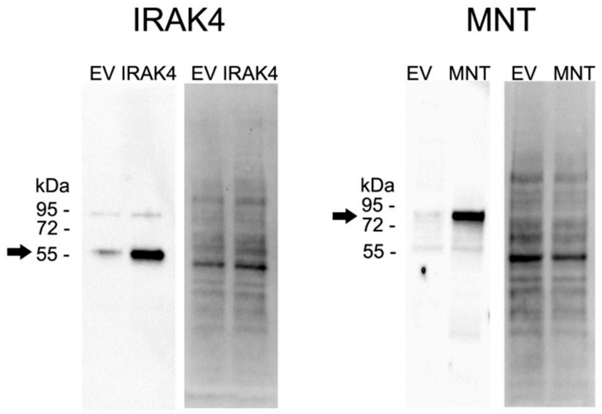

Figure S7. Uncropped western blot results for IRAK4 and MNT as presented in Figure 5. Arrows indicate the protein of interest.

```
5′GATATCGCCCTGATCAAGAGCGAAGAGGGCGAGAAAATGGTGCTTGAGAATAACTTCTTCGTCGAGA
CCATGCTCCCAAGCAAGATCATGCGGAAACTGGAGCCTGAGGAGTTCGCTGCCTACCTGGAGCCATTCA
AGGAGAAGGGCGAGGTTAGACGGCCTACCCTCTCCTGGCCTCGCGAGATCCCTCTCGTTAAGGGAGGCA
AGCCCGACGTCGTGCAAATTGTCCGCAACTACAACGCCTACCTTCGGGCCAGCGACGATCTGCCTAAGA
TGTTCATCGAGTCCGACCCTGGGTTCTTTTCCAACGCTATTGTCGAGGGAGCTAAGAAGTTCCCTAACA
CCGAGTTCGTGAAGGTGAAGGGCCTCCACTTCAGCCAGGAGGACGCTCCAGATGAAATGGGTAAGTACA
TCAAGAGCTTCGTGGAGCGCGTGCTGAAGAACGAGCAGTAATTCTAGGCGATCGCTCGAG3′
```

Figure S8. Sequence of the minigene used to generate a luciferase reporter vector without the putative miR-378a-3p binding site in the Renilla gene. The miR-378a seed sequence in shown in green, with the mutated nucleotides in red.

Table S1. Small RNA sequencing read summary.

Sample	Total after Trimming		Mapping to Human Genome			Mapping to miRbase 21		
	Reads	Collapsed	Reads	Percentage	Collapsed	Reads	Percentage	Collapsed
ST486	12,728,371	679,193	9,215,947	72.4%	477,577	7,951,451	62.5%	19,074
CA46	17,810,149	193,500	15,429,749	86.6%	45,386	13,035,709	73.2%	17,595
DG75	5,553,807	295,796	4,542,877	81.8%	192,986	3,267,634	58.8%	16,337
Ramos	9,001,167	566,396	6,671,654	74.1%	390,175	4,040,003	44.9%	14,824
GC-1	9,234,697	357,071	6,993,299	75.7%	176,567	5,541,436	60.0%	15,998
GC-2	9,637,128	620,514	5,225,051	54.2%	389,723	2,534,968	26.3%	13,262
GC-3	6,758,291	524,000	4,660,685	69.0%	284,438	3,563,084	52.7%	15,879

Table S2. Taqman miRNA assays used for qRT-PCR validation.

No.	miRNA	Catalog No.	Sequence
1	miR-378a-3p	2243	5′-ACUGGACUUGGAGUCAGAAGG-3′
2	miR-28-5p	0411	5′-AAGGAGCUCACAGUCUAUUGAG-3′
3	miR-155-5p	2623	5′-UUAAUGCUAAUCGUGAUAGGGGU-3′
4	miR-363-3p	1271	5′-AAUUGCACGGUAUCCAUCUGUA-3′
5	miR-222-3p	2276	5′-AGCUACAUCUGGCUACUGGGU-3′
6	miR-221-3p	1134	5′-AGCUACAUUGUCUGCUGGGUUU-3′
7	miR-30a-5p	0417	5′-UGUAAACAUCCUCGACUGGAAG-3′
8	miR-28-3p	2446	5′-CACUAGAUUGUGAGCUCCUGGA-3′

Table S3. Oligos for cloning miR-378a-3p binding sites from selected genes, wild type and with mutations in miR-378a-3p seed. Green letters indicate miR-378a-3p binding sites and red letters indicate the mismatches.

NO.	Gene	Sense(S)/ Anti-sence(AS)	5′ to 3′
1	FOXP1	FOXP1-site1-MBS-S	TCGAGAAGGGCCCCTGTCCTTAGTGACAACAGCCAACCACAGTCCAGATTTTGACCAT
		FOXP1-site1-MBS-AS	GGCCATGGTCAAAATCTGGACTGTGGTTGGCTGTTGTCACTAAGGACAGGGGCCCTTC
		FOXP1-site1-MBS-mut-S	TCGAGAAGGGCCCCTGTCCTTAGTGACAACAGCCAACCACACTGCTGATTTTGACCAT
		FOXP1-site1-MBS-mut-AS	GGCCATGGTCAAAATCAGCAGTGTGGTTGGCTGTTGTCACTAAGGACAGGGGCCCTTC
		FOXP1-site2-MBS-S	TCGAGCAATGGAGCATACCAACAGCAACGAGAGTGACAGCAGTCCAGGCAGATCTCCT
		FOXP1-site2-MBS-AS	GGCCAGGAGATCTGCCTGGACTGCTGTCACTCTCGTTGCTGTTGGTATGCTCCATTGC
2	IRAK4	IRAK4-MBS-S	TCGAAATCTTGAACAAAGCTATATGCCACCTGACTCCTCAAGTCCAGAAAATAAAAGT
		IRAK4-MBS-AS	GGCCACTTTTATTTTCTGGACTTGAGGAGTCAGGTGGCATATAGCTTTGTTCAAGATT
		IRAK4-MBS-mut-S	TCGAAATCTTGAACAAAGCTATATGCCACCTGACTCCTCAACTGCTGAAAATAAAAGT
		IRAK4-MBS-mut-AS	GGCCACTTTTATTTTCAGCAGTTGAGGAGTCAGGTGGCATATAGCTTTGTTCAAGATT
3	JPX	JPX-MBS-S	TCGAGTTGCAAGGCGTCCGAAGTATGAGTCCACTAACAAAAGTCCAGAAACTCGCCAGT
		JPX-MBS-AS	GGCCACTGGCGAGTTTCTGGACTTTTGTTAGTGGACTCATACTTCGGACGCCTTGCAAC
		JPX-MBS-mut-S	TCGAGTTGCAAGGCGTCCGAAGTATGAGTCCACTAACAAAAGACGACAAACTCGCCAGT
		JPX-MBS-mut-AS	GGCCACTGGCGAGTTTGTCGTCTTTTGTTAGTGGACTCATACTTCGGACGCCTTGCAAC
4	MNT	MNT-MBS-S	TCGACATCGGGGGCCTGCAAATCTAGTGCCGAATGACTATGTCCAGATTGGTGACGAT
		MNT-MBS-AS	GGCCATCGTCACCAATCTGGACATAGTCATTCGGCACTAGATTTGCAGGCCCCCGATG
		MNT-MBS-mut-S	TCGACATCGGGGGCCTGCAAATCTAGTGCCGAATGACTATGACGACATTGGTGACGAT
		MNT-MBS-mut-AS	GGCCATCGTCACCAATGTCGTCATAGTCATTCGGCACTAGATTTGCAGGCCCCCGATG
5	BCR	BCR-site1-MBS-S	TCGAGGCCACCTGAGGGCGCCCCAAGCCAGTTCATCTCGGAGTCCAGGCCTGGCCCTG
		BCR-site1-MBS-AS	GGCCCAGGGCCAGGCCTGGACTCCGAGATGAACTGGCTTGGGGCGCCCTCAGGTGGCC
		BCR-site2-MBS-S	TCGAACCTCACCTCCAGCGAGGAGGACTTCTCCTCTGGCCAGTCCAGCCGCGTGTCCC
		BCR-site2-MBS-AS	GGCCGGGACACGCGGCTGGACTGGCCAGAGGAGAAGTCCTCCTCGCTGGAGGTGAGGT
6	CISH	CISH-MBS-S	TCGAGCGAGCCAACCCCACCTCTATGCCCTGAGCCCTGGTAGTCCAGAGACCCCAACT
		CISH-MBS-AS	GGCCAGTTGGGGTCTCTGGACTACCAGGGCTCAGGGCATAGAGGTGGGGTTGGCTCGC
7	MYCBP	MYCBP-MBS-S	TCGAGTGCCTCTTCTAGTCACAAGTTTGTTTTTGGAGGGGGTCCAGAAGATCATTCCC
		MYCBP-MBS-AS	GGCCGGGAATGATCTTCTGGACCCCCTCCAAAAACAAACTTGTGACTAGAAGAGGCAC

8	TUBA1C	TUBA1C-MBS-S	TCGACGTGAGTGCATCTCCATCCACGTTGGCCAGGCTGGTGTCCAGATTGGCAATGCC
		TUBA1C-MBS-AS	GGCCGGCATTGCCAATCTGGACACCAGCCTGGCCAACGTGGATGGAGATGCACTCACG

Publisher's Note: MDPI stays neutral with regard to jurisdictional claims in published maps and institutional affiliations.

© 2020 by the authors. Licensee MDPI, Basel, Switzerland. This article is an open access article distributed under the terms and conditions of the Creative Commons Attribution (CC BY) license (http://creativecommons.org/licenses/by/4.0/).
